# Reply to: Animal magnetic sensitivity and magnetic displacement experiments

**DOI:** 10.1038/s42003-024-06270-x

**Published:** 2024-05-27

**Authors:** Will T. Schneider, Joe Wynn, Florian Packmor, Oliver Lindecke, Richard A. Holland

**Affiliations:** 1 School of Natural Sciences, Bangor University, Bangor, Gwynedd LL57 2UW UK; 2 Institute of Avian Research, 26386 Wilhelmshaven, Germany; 3 Lower Saxon Wadden Sea National Park Authority, 26382 Wilhelmshaven, Germany; 4 Institute of Biology and Environmental Sciences, University Oldenburg, 26111 Oldenburg, Germany

**Keywords:** Animal behaviour, Animal migration

**replying to**: K. J. Lohmann et al. *Communications Biology* 10.1038/s42003-024-06269-4 (2024)

In their matters arising article, Lohmann et al. propose that animals have a higher sensitivity to geomagnetic cues than we assumed in our original article. Based on this they re-assert the feasibility of a magnetic map used by animals to navigate. This second point represents a misunderstanding of our conclusions. We do not rule out magnetic maps as a possible tool for animal navigation, or that the presence of multiple locations means that animals are not responding to changes in the magnetic field. Nor do we suggest that virtual magnetic displacements are a flawed technique. Our aim is not to disparage previous work, rather, we highlight the need for caution in how virtual magnetic displacement experiments are designed and in how results are interpreted. We suggest caution because we have found that many virtual displacement studies do not appear to consider the presence of multiple possible locations that have the same magnetic cues. The onus here should be on the authors of studies to consider all possible locations and provide evidence and/or discussion of how the presence of possible locations may be interpreted by their test subject. The tool we present in our original article will enable researchers to visualise possible locations to help them to make these necessary considerations. The lack of evidence for how sensitive animals are to magnetic cues is certainly a major driver in how widespread the multiple locations with the same magnetic cues may be.

This leads to Lohmann et al.’s issue with the sensitivity assumptions that we made. We agree that this poses an interesting and timely question: How sensitive are animals to the Earth’s magnetic field? To address this, we here raise three topics we think may be of relevance: a) How sensitive can we reasonably expect animals to be to the Earth’s magnetic field? b) Is sensitivity in a stationary virtual displacement equal to sensitivity when free-moving? And c) how do we interpret the current literature to determine sensitivity?

**Fig. 1:**
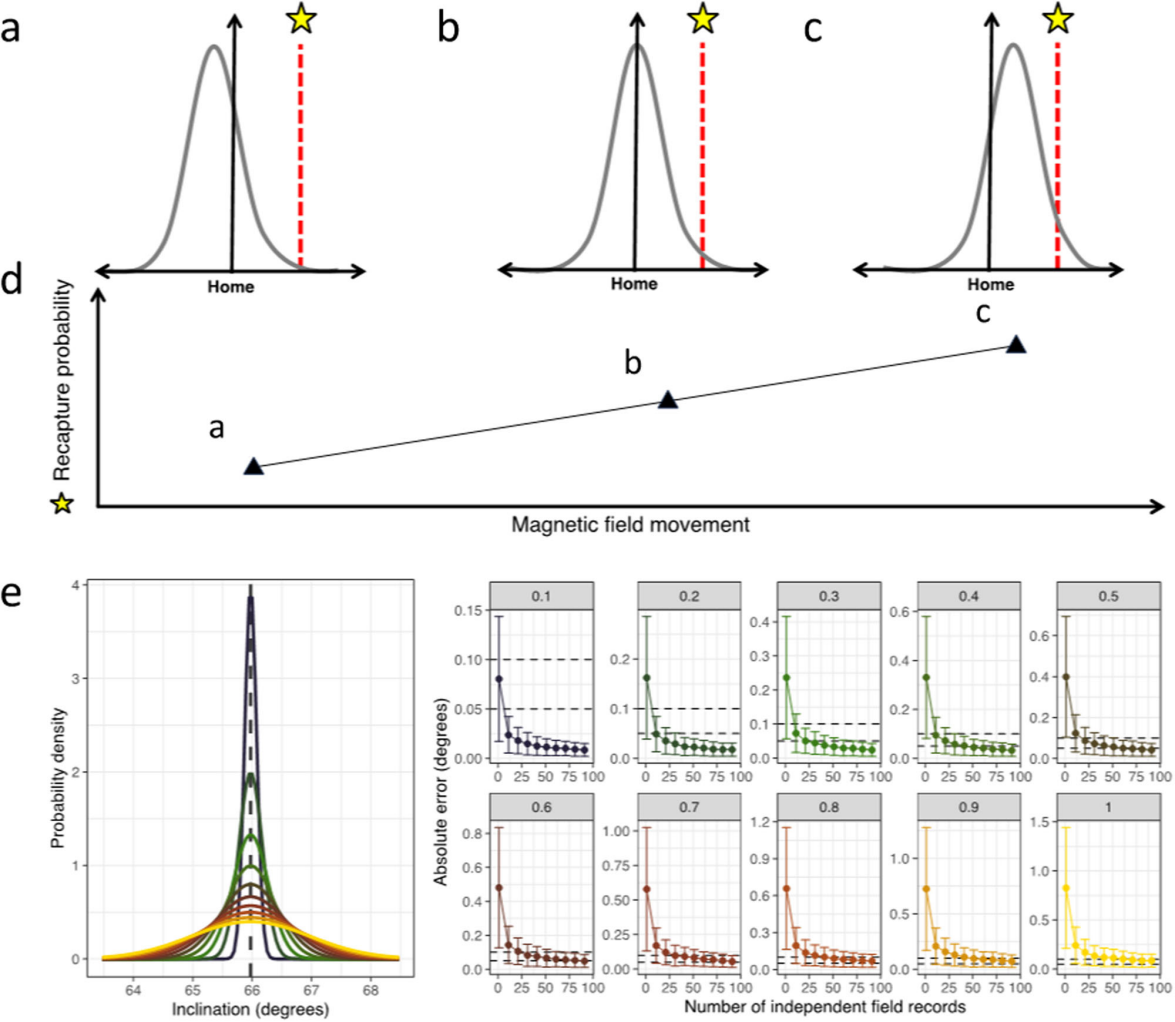
Magnetic secular variation and philopatry. **a**–**d** A schematic demonstrating how slight magnetic secular variation might cause changes in distribution even in animals with low-resolution magnetoreceptors. In Case **a**, the magnetic field has shifted left slightly, causing a drop in recovery probability at the starred location even though most animals return to the natal site (owing to the tight distribution caused by low magnetic sensitivity). Case **b** is intermediate, whilst Case **c** shows the opposite phenomenon; the field has shifted right, and a response can be recorded at the starred site even though the sensitivity of any given animal is very low. **e** A simulation showing how even noisy sensors can achieve remarkably accurate philopatry (<0.05 degrees) through repeated sampling of the magnetic field. Each point represents 100 simulations where the magnetic field is sampled *n* times with both sensor error and local secular variation. Whiskers represent standard deviation. Magnetic data is derived from the Hartland Magnetic Observatory (UK).

In their response, Lohmann et al. interpret our assumed sensitivity—0.5° inclination and 200 nT intensity—as coarser than that which might be expected of a wild animal. How, then, can an animal’s magnetic sensitivity be assessed? Lohmann et al. use the range of values required to elicit a measurable response as their criterium. Whilst this might give some idea of sensitivity, it may be an imperfect approach for several reasons. Firstly, very coarse-grain sensors can be tested for with small variation if the sample size is sufficient. For example, Lohmann et al. cite two studies that used tiny amounts of year-on-year secular variation (c.0.02–0.05 degrees) to suggest that philopatry in birds is magnetically determined^1,2^. These studies, however, have enormous sample sizes (*n* = 2996 and *n* = 17,799), and hence, the effects reported might reflect shifts in the mean position of a tight but long-tailed distribution. This way, even a 0.01-degree shift in inclination might be detected hundreds of kilometres away since the long-tailed distribution’s movement might be minimal (since sensitivity is low), yet a statistical trend might be observed given the large sample size (see Fig. 1).

Secondly, sensitivity assessed via philopatric accuracy might differ markedly from the actual physical sensitivity of a system. In much the same way that the winning guess of ‘number of jelly beans in the jar’ is often astonishingly accurate—despite each participant’s ability to count beans being relatively poor—repeated estimations of position using a noisy magnetoreceptor can give the impression of accuracy far beyond that predicted by its physical sensitivity. Indeed, a simple simulation using normally distributed noise suggests that a sensor with a sensitivity of 0.6° can achieve a functional sensitivity of 0.05° by measuring the magnetic field instantaneously once a day for 100 days (an increase in accuracy of more than an order of magnitude; see Fig. 1). This could explain the magnetic imprinting observed in birds, where a protracted post-fledging period is almost guaranteed. Such an ability would not, however, explain responses to virtual displacement; these experiments lack the repeated sampling required to improve sensitivity using central tendency.

Thirdly, during a virtual magnetic displacement, the subject animal experiences a constant magnetic field. Importantly, there is no opportunity for the animal to move and experience alterations in the field that may allow it to take multiple readings and gain an impression of gradients of change and, therefore, discriminate between minimal signals. If one imagines a test in which someone puts their hand into a bath of warm water for several minutes, removes it, waits several hours, then puts the hand into a new bath of water with a difference of 1 °C, and then determines which bath was hotter—it would be an extremely difficult test. If, instead, there was a single bath of water, and in one end it was 1 °C hotter than the other, then by moving the hand back and forth in the bath it would be relatively easy to determine which is the hotter end. In both scenarios the sensitivity of the hand is the same, but the test in the first is far more challenging. The first (difficult) scenario is comparable to the opportunity provided to an animal when it has been virtually magnetically displaced and is required to determine the parameters of the field. In contrast, the second scenario allows for the possibility of sensing gradients of change. All of the citations that Lohmann et al. provide as evidence for higher sensitivity values than those we used are relevant only to animals that were allowed to experience gradients of change of magnetic fields^1–10^. Therefore, they are not relevant to how sensitive an animal may be to a virtual magnetic displacement.

**Fig. 2:**
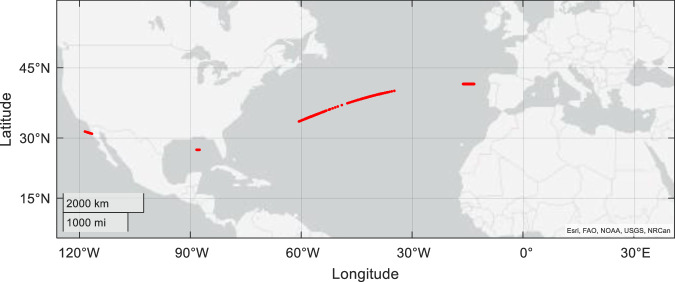
Multiple possible virtual displacement locations with high sensitivity assumed. Red dots indicate all the locations that share the same magnetic parameters (magnetic total intensity = 45083 nT, magnetic inclination = 56.42 degrees) with a sensitivity assumption of ±20 nT for magnetic intensity, and ±0.05 degrees for magnetic inclination. Magnetic values obtained from the IGRF for May 2020.

It is, then, unclear from the literature what our expectation of sensitivity to magnetic displacement is expected to be for any taxa. This, in turn, increases the utility of the tool presented in our original manuscript since, given this ambiguity, it is perhaps better to be safe than sorry. Nonetheless, whilst we believe that the parameters presented in our original manuscript represent a ‘best case’ for magnetic sensitivity (it is hard to imagine, based on other sensory inputs, a sub-1-degree sensitivity, or sensitivity below the daily secular variation of the geomagnetic field), it is perhaps worth considering how virtual displacement might function under an assumption of extremely high sensitivity (far higher than we believe there is evidence for). To that end, our tool demonstrates that even in such instances many possible locations can still exist spanning a very large geographic area. For example, Fig. 2 shows that even if animals are sensitive to 0.05° of inclination and 20 nT of intensity, there are locations spanning the width of the mid-Atlantic Ocean (locations, for example, that are highly relevant to the movements of loggerhead turtles), and in the Gulf of Mexico and Western Pacific Ocean, that all have the same magnetic values (red dots in Fig. 2). We might conclude, therefore, that (a) an understanding of absolute magnetoreceptive sensitivity requires further research going forward using a different paradigm to behavioural responses to changes in the magnetic field and, (b) that irrespective of sensitivity, extreme care must be taken when conceptualising and interpreting behavioural responses to virtual displacement.
